# Topological Effects of Bottlebrush Copolymer on Their Assembly at the Water/Air Interface

**DOI:** 10.1021/acs.langmuir.6c00890

**Published:** 2026-05-28

**Authors:** Shubhadeep Nag, Nazrul Islam, Titilayo Deborah Oluwole, Jimmy Lawrence, Yaxin An

**Affiliations:** Department of Chemical Engineering, 5779 Louisiana State University, Baton Rouge, Louisiana 70803, United States

## Abstract

Amphiphilic bottlebrush copolymers offer exceptional tunability through their grafted side-chain architectures, yet the molecular principles linking topology to interfacial assembly remain incompletely understood. Here, we use coarse-grained molecular dynamics simulations to investigate the influence of four topological design parameters: block length (*l*), side-chain dispersity (*Đ*), grafting density (*f*), and the backbone length (*N*_bb_) on the assembly of PEG–PS (PEG: poly­(ethylene glycol), PS: polystyrene) bottlebrush copolymers at the water/air interface. The assembled structures are characterized by three methods: the interfacial area per molecule, the average radius of gyration of PEG–PS chains, and the hydration of PEG. By varying each parameter while keeping overall hydrophilic and hydrophobic content fixed, we identify the distinct physical mechanisms by which topology dictates molecular organization at the interface. Increasing block length enhances PEG–PS segregation, producing a few-percent reduction in chain dimensions and a tens-of-percent decrease in the interfacial area per molecule, making block length the strongest determinant of packing. In contrast, the dispersity and grafting density of PEG and PS induces only minor changes (∼5–10%) in the interfacial area per molecule at the studied range. The orientational distributions of side chains relative to the interface further reveal that alternating architectures exhibit strongly separated orientations of hydrophobic and hydrophilic segments, while diblock architectures show more overlapping orientations, which govern chain extension and interfacial packing. As the *N*_bb_ increases, the orientation of side chains in the diblock polymer becomes more constrained. The hydration of hydrophilic chains varies differently with the topological design parameters. The grafting density significantly impacts the hydration of PEG side chains, while the block length and the dispersity play a minor role in the local water structures. The simulations provide molecular-level insights into the experimentally observed differences in surface packing and the corresponding trends in interfacial tension of bottlebrush copolymers. This work establishes a topology–assembly relationship and provides molecular-level design principles for bottlebrush copolymers.

## Graphical abstract

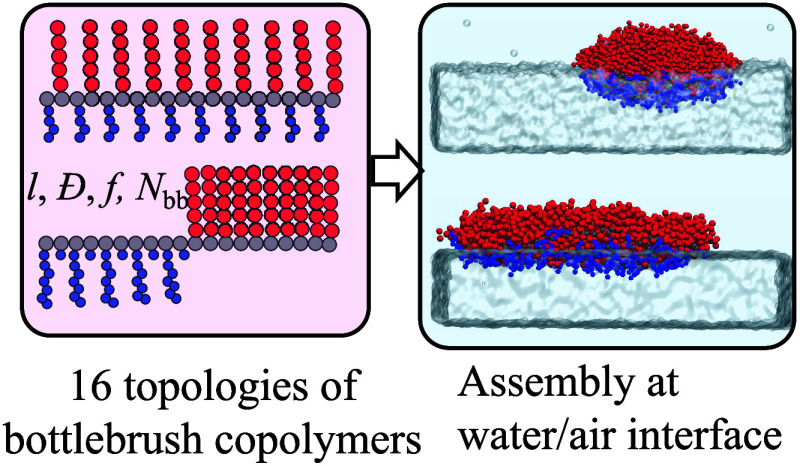

## Introduction

Bottlebrush polymers are a class of linear macromolecules highly grafted with polymeric side chains. The densely grafted side chains greatly enhance the effective stiffness of the backbone; thus, the bottlebrush polymers are less likely to be entangled, leading to unique mechanical and rheological properties.^1–7^ The topology of bottlebrush polymers is crucial in governing their properties. A variety of topologies have been reported, e.g., uniform,^8,9^ tadpole,^10^ dumbbell,^11^ and tapered structures^12^ by varying the side-chain or backbone lengths. The thermodynamic properties, such as glass transition temperatures, conformational entropy, etc., can be significantly changed by tuning the topologies of bottlebrush polymers.^8,9,13–15^ Moreover, properties that are traditionally correlated in linear polymers can be decoupled in bottlebrush polymers by controlling the backbone and side-chain dynamics separately.^13,16^ This presents opportunities to tailor various properties of bottlebrush polymers independently, which is not achievable by linear polymers.

Bottlebrush copolymers, where side chains comprise two or more different polymers, provide richer topological diversity than homogeneous bottlebrush polymers with identical side chains.^17,18^ Amphiphilic bottlebrush polymers are a class of copolymers consisting of both hydrophilic and hydrophobic polymers as side chains.^19–22^ They can assemble into ordered microscopic morphologies, which crucially determine their properties in applications such as drug delivery,^23,24^ 3-D printing,^25^ and photonics.^26^ The molecular architectures are therefore essential to their assembly formation in the bulk and at the interface.^27^ Hsieh et al. investigated the effects of the side-chain/backbone length on the xylene-water interfacial dilatational rheology of PEO–PBA (PBA: poly­(*n*-butyl acrylate)) bottlebrush polymers.^28^ The authors reported the strong anchoring of bottlebrush polymers with long PEO side chains at the interface, which hinders the segment detachment from the interface. Besides the side-chain/backbone lengths, the sequencing of hydrophilic and hydrophobic side chains impacts their assembled structures. Oluwole et al. compared the interfacial tensions and interfacial assembly kinetics of diblock and alternating bottlebrush copolymers of PS–PEO (PS: polystyrene) and PDMS–PEO (PDMS: poly­(dimethylsiloxane)).^29^ The diblock copolymers exhibit more reduction in the interfacial tension than the alternating copolymers, due to the higher packing density of the diblock bottlebrush copolymers. Jiang et al. investigated the assembly of block and gradient bottlebrush PS–PLA (PLA:polylactide) copolymers in the melt.^30^ In block bottlebrush PS–PLA copolymers, PS and PLA polymers are separated as two blocks with an abrupt change from PS to PLA branches along the linear backbone, while they are mixed in gradient bottlebrush copolymers with the composition of PLA gradually increasing along the backbone. The block bottlebrush copolymers pack into lamellar microstructures while gradient bottlebrush copolymers form cylindrical morphologies. Seong et al. investigated the assembly of bottlebrush copolymers of PDMS–PEO with random and block architectures, i.e., BRCP (bottlebrush random copolymer) and BBCP (bottlebrush block copolymer) at the water/toluene interface.^31^ The interface tension of BRCP increases more significantly as the backbone length increases, while that of the BBCP increases slightly. The large topological diversity presents an opportunity for tuning their assembly in polymer melts, in solutions, and at the surfaces/interfaces. On the other hand, it challenges the fundamental understanding of how the topology of bottlebrush polymer amphiphiles governs their assembly. These experimental studies primarily rely on macroscopic observables that cannot provide a molecular-level understanding of the respective chain conformations, orientations, or hydration.

Recent molecular simulation studies, including both all-atom/coarse-grained molecular dynamics (MD) and dissipative particle dynamics (DPD), provide microscopic insight into the assembly of surfactants and predict macroscopic properties.^30,32–36^ All-atom MD simulations are computationally costly for simulating the assembly of macromolecules. Coarse-grained simulations, which represent atomistic groups by coarse-grained beads, are computationally efficient and thus attract lots of interest in the assembly of macromolecules.^37–42^ Wessel et al. performed coarse-grained simulations with an implicit solvent to study the self-assembly of bottlebrush amphiphiles by varying the sequence of solvophilic and solvophobic segments along the backbone with a fixed total number of beads.^43^ They observed a transition from spherical micelles to cylindrical micelles and bilayers governed by critical packing parameters. Other coarse-grained simulations have further characterized the conformations and dynamics of neutral bottlebrush polymers in dilute solutions,^44^ melts,^45^ and responsive systems using machine learning,^46^ revealing how grafting density and side-chain length control persistence length, radial monomer distributions, and temperature-dependent coil–globule transitions through scattering validation and single-chain analysis.^47^ Recently, Ogbonna et al. combined experiments with MD simulations to investigate the effect of side-chain dispersity on bottlebrush assembly at the air–water interface, showing that discrete side chains significantly alter interfacial packing and phase behavior.^48^ Though these studies establish structure–property relationships for bottlebrush polymers in bulk and solution environments,^43,44,49^ molecular-level insights into assembly of bottlebrush copolymer at the liquid–air interface need to be explored.

DPD simulations have also been widely employed to investigate bottlebrush polymers in solutions and near interfaces. Wang et al. demonstrated that bottlebrush polymers self-assemble into a variety of microstructures, including spherical, cylindrical, and lamellar phases, depending on concentration and side-chain architecture, and further analyzed their interactions with lipid membranes.^50^ Similarly, Dai et al. examined the adsorption and deformation of bottlebrush polymers at phospholipid membranes, highlighting the role of polymer–membrane interactions and chain architecture in governing structural response.^51^ More recently, Bugaeva et al. studied amphiphilic bottlebrushes at liquid interfaces using mesoscopic simulations, showing that adsorption and compression can induce morphological transitions from isolated aggregates to continuous monolayers and percolated structures.^52^ However, these studies predominantly focus on bulk, solution, or membrane-associated systems, and a systematic molecular-level understanding of bottlebrush copolymer assembly at explicit liquid–air interfaces remains lacking. In particular, the combined effects of controlled sequence architectures (e.g., alternating, multiblock, and diblock), discrete bimodal side-chain distributions, and explicit solvent representation at the water–air interface have not been explored. These factors are of particular interest because they directly control chain packing, interfacial orientation, and lateral spreading,^22,53,54^ thereby governing the resulting interfacial morphology and macroscopic properties such as surface coverage and tension reduction.

In this work, we aim to address the fundamental questions of how the topology of bottlebrush polymer amphiphiles impacts their assembly at the water/air interface by carrying out a comprehensive set of coarse-grained molecular dynamics simulations. We investigate the topological effects of bottlebrush polymers with hydrophilic PEG (poly­(ethylene glycol)) and hydrophobic PS side chains, which have been synthesized in experiment.^29^ Specifically, the topological parameters, including the sequencing of PEG/PS side chains (i.e., the block length of PEG/PS), the side-chain dispersity (*Đ*), and the grafting density (*f*), are altered to construct bottlebrush copolymers. Coarse-grained Martini models are utilized to represent bottlebrush copolymers and study their assembly at the water/air interface. We calculate the surface area covered by the assembled clusters of bottlebrush copolymers, the average radius of gyration of a bottlebrush polymer chain in the assembly, and the hydration of PEG. The outcome of this study will guide the topological design of bottlebrush polymer surfactants, paving the way to their chemical and biological applications.

## Methods

### Coarse-Grained Model Construction

All bottlebrush polymer amphiphiles in this study were modeled using the Martini 2 coarse-grained (CG) force field.^39,55–58^ The backbone of each bottlebrush polymer was represented using C1 beads (nonpolar aliphatic units), with a backbone length fixed at 20 beads; backbone stiffness was maintained through a harmonic angle potential applied between consecutive backbone beads. The hydrophilic side chains, PEG, are represented by *n* EO beads (ethylene oxide groups), and *n* defines the degree of polymerization. The hydrophobic side chains are composed of PS, where each styrene monomer was mapped to four SCY beads (aromatic hydrophobic units). The PEG and PS Martini 2 models were adopted from established literature parametrizations, where the PEG and PS models have been shown to be transferable and mutually compatible for block copolymer systems^59,60^ The bonds and angles between the beads are modeled with weak harmonic potentials.^61^ Note that the angle potential is of the cosine type. The corresponding parameters are listed in Table 1, and the nonbonded LJ parameters are listed in Table 2. Here, nonbonded LJ refers to the Lennard–Jones potential describing effective nonbonded interactions between coarse-grained beads in the Martini model. The self- and cross nonbonded LJ interaction parameters for the bead types (SCY, EO, and C1) along with their interaction with water beads were obtained from the Martini 2 literature.^59,60^ In this framework, the interaction strengths reflect bead polarity classes according to Martini guidelines and are calibrated by the Martini developer to reproduce thermodynamic partitioning behavior between polar and nonpolar environments, such as the transfer free energies of chemical groups between water and hydrophobic phases. As a result, the relative magnitudes of the Lennard–Jones well depths describe the balance between hydrophilic and hydrophobic interactions rather than direct pairwise chemical affinities. A representative coarse-grained model of a bottlebrush polymer amphiphile with alternating PEG (blue) and PS (red) side chains grafted onto a C1 backbone (gray) is shown in Figure 1(a).

**Figure 1:**
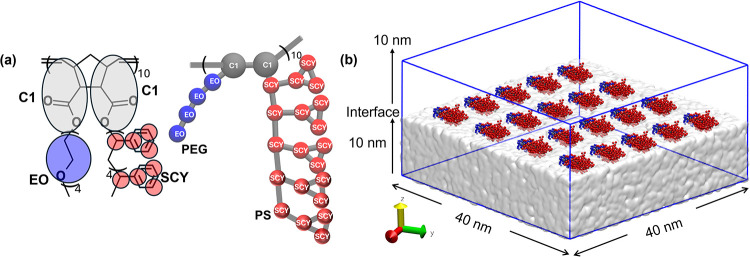
Chemical structures and coarse-grained representations of the (a) bottlebrush copolymer with alternating PEG (blue) and PS (red) side chains grafted onto a polymer backbone (gray) are shown here. In (b), we show the initial configuration of 20 such polymers on the surface of 40 nm × 40 nm × 10 nm of water. Along the *z*-axis, there is a 10 nm air medium above the interface.

**Table 1:** Bond and Angle Interaction Parameters between Polymer Beads

Bond Parameters		
bond type	*k*_b_ (kcal/mol/Å^2^)	*b*_0_ (Å)
SCY–SCY	1.4937	4.7
SCY–C1	1.4937	4.7
C1–C1	1.4937	4.7
C1–EO	1.4937	4.1
EO–EO	8.3652	3.2

angle type	*k*_θ_ (kcal/mol)	θ_0_ (deg)
EO–EO–EO	5.975	135
C1–C1–SCY	2.9875	180
C1–SCY–SCY	2.9875	180
SCY–SCY–SCY	2.9875	180
C1–EO–EO	2.9875	180
C1–C1–EO	2.9875	180

**Table 2:** Non-Bonded LJ Interaction Parameters of Polymer and Water

	Polymer–Polymer Interaction Parameters	
bead–bead	*R*_min_ (Å)	ϵ (kcal/mol)
SCY-SCY	4.826	0.6274
SCY-C1	5.275	0.8365
SCY-EO	4.826	0.5442
C1-EO	5.275	0.6046
EO-EO	4.826	0.6094
C1–C1	5.275	0.8365

	Polymer–Water Interaction Parameters	
bead–bead	*R*_min_ (Å)	ϵ (kcal/mol)
SCY-P4/BP4	5.275	0.4779
C1–P4/BP4	5.275	0.4779
EO-P4/BP4	5.275/4.826	0.8365/0.7528

	Water–Water Interaction Parameters	
bead–bead	*R*_min_ (Å)	ϵ (kcal/mol)
P4–P4	5.275	1.1950
BP4-P4	6.398	1.3384
BP4-BP4	5.275	1.1950

The degree of polymerization of PEG side chains is fixed at 4, and each PS side chain contains 5 styrene monomers (20 SCY beads) in the diblock/multiblock and alternating architectures (see Figures 1(a) and 2). The block length of the hydrophilic PEG and hydrophobic PS segments is defined here as the number of consecutive side chains of the same type grafted along the backbone, distinguishing it from the backbone length and individual side-chain length. Besides the uniform side-chain lengths of PEG/PS, the side-chain dispersity (*Đ* > 1) is also varied to study its effects on the assembly. It is to note that the term dispersity in our work does not refer to a continuous molecular weight distribution;^62^ instead, the side-chain heterogeneity is introduced through discrete mixtures of two predefined chain lengths, resulting in a bimodal distribution. For PEG, dispersity values of 1.06, 1.25, and 1.53 are obtained by mixing side chains with degrees of polymerization 5/3, 6/2, and 7/1, respectively (see Figure S1 of the Supporting Information (SI)). Similarly, the dispersity *Đ* of PS side chains is increased from 1.04 to 1.36 by using two different side-chain lengths, 6/4 and 8/2 (Figure S2 of the SI). In this way, the dispersity of side chains is varied; however, the total number of EO/SC beads is fixed at 40 and 200, respectively. The grafting density *f* is defined as the fraction of backbone beads that are grafted with a side chain. Thus, *f* = 1 corresponds to grafting at every backbone bead, while lower values (e.g., *f* = 0.1) correspond to one side chain per ten backbone beads. In our work, *f* was varied by altering the frequency with which PEG and PS side chains were attached to the backbone (e.g., grafting every backbone bead, every second bead, or mixed sequences). Side-chain dispersity *Đ* > 1 was implemented using a heterogeneous set of fixed side-chain lengths across different grafting sites such that each bottlebrush chain contained side chains of predetermined lengths rather than lengths drawn from a continuous distribution (see Figures S1/S2 of the SI). The aqueous environment was modeled using P4 coarse-grained water beads, with around 10% BP4 antifreeze beads included to prevent freezing.

**Figure 2:**
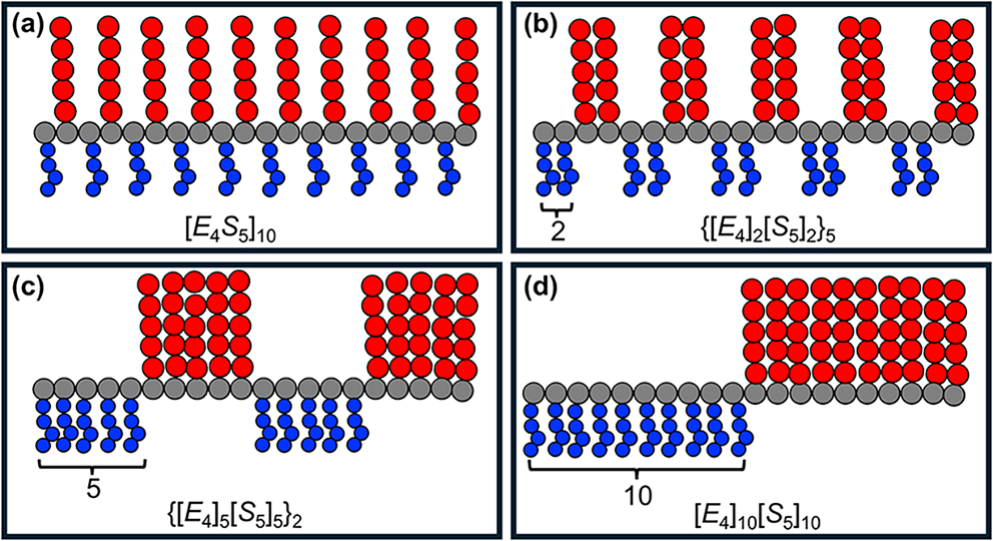
Schematic representation of the four block-length architectures studied: (a) alternating sequence [*E*_4_ *S*_5_]_10_ (block length 1); (b) short-block topology {[*E*_4_]_2_[*S*_5_]_2_}_5_ (block length 2); (c) intermediate-block topology {[*E*_4_]_5_[*S*_5_]_5_}_2_ (block length 5); (d) diblock architecture [*E*_4_]_10_[*S*_5_]_10_ (block length 10). PEG side chains are shown in blue, PS in red, and backbone beads in gray. Note that each red circle represents one styrene monomer (four SCY beads in Figure 1a). These schematics illustrate the sequence patterning differences that give rise to the conformational and packing behavior analyzed in Figure 3.

### System Setup

A rectangular water box of dimensions 40 nm × 40 nm × 10 nm was first generated, containing 128,000 P4 beads and 10,000 BP4 beads; to create a water/air interface, the simulation box was extended in the *z*-direction to a final size of 20 nm, introducing an air slab above the aqueous layer, thereby creating a well-defined water–air interface. The air/water interface is defined using the Gibbs dividing surface, conceptually identified from the variation of the water density profile along the *z*-direction, where the density transitions from its bulk liquid value to the vapor phase. In the present work, the interface is not explicitly tracked over time; instead, the analysis is performed in a coordinate framework where the interfacial region remains stable. The bottlebrush polymers are localized at this interface, with hydrophilic PEG (EO) segments preferentially oriented toward the aqueous phase, while hydrophobic PS (SCY) segments extend toward the air phase. The backbone remains near the interfacial region, connecting the two domains. A total of 20 bottlebrush polymer amphiphiles were placed at the water/air interface, in a 4 × 5 lateral grid, with an initial center-of-mass separation of approximately 4 nm between adjacent molecules. The initial configuration was energy-minimized to remove steric overlaps (see Figure 1(b)). Periodic boundary conditions were imposed in all three directions.

Additional simulations were performed to examine the effects of increased backbone length and surface concentration. For the backbone length study (*N*_bb_ = 40), systems were simulated using the same interfacial dimensions (400 × 400 × 200 Å^3^) as the original systems. To probe higher surface concentration, equilibrated aggregates from the *N*_bb_ = 20 systems were placed in a reduced interfacial area (250 × 250 × 200 Å^3^), while keeping the box length along the *z*-direction unchanged. The corresponding system contains 4736 BP4 water beads and 47364 P4 beads, ensuring consistent solvent representation under increased confinement.

### Simulation Details

All simulations were performed using NAMD 2.14 ^63^ in the NVT ensemble at 310 K; temperature was controlled using a Langevin thermostat with a damping coefficient of 1 ps^–1^; the integration time step was 20 fs, consistent with standard Martini 2 simulations. Standard Martini 2 nonbonded parameters were used: Lennard-Jones interactions employed a cutoff of 1.2 nm with switching starting at 0.9 nm; Neighbor lists were updated every 10 integration steps. Each system was equilibrated for 100 ns and subsequently simulated for a production run of 1 μs. In our work, we systematically computed radial distribution functions (RDFs), the radius of gyration, *R*_g_, the interfacial area per molecule (*A*) of the polymers, and the orientational distributions of side chains relative to the interface (angle with respect to the interface). The details of the RDF, *R*_g_, and interfacial area (*A*) analyses are provided in the Analysis of the SI.

## Results and Discussion

Bottlebrush polymer amphiphiles exhibit a rich diversity of topological architectures, and this diversity provides opportunities for tailoring interfacial assembly while simultaneously obscuring the underlying topology–property relationships. To elucidate the fundamental principles by which topology governs assembly at the water/air interface, we systematically vary four key structural parameters:

1. The block length of the hydrophilic PEG and hydrophobic PS segments,
2. The side-chain dispersity *Đ*, and
3. The grafting density *f*.
4. The backbone length *N_bb_.*

Throughout all simulations, the total number of EO beads per polymer is fixed at 40; each PS side chain contains five styrene monomer units (represented by four SCY beads per monomer), and each bottlebrush polymer contains ten PS side chains. We evaluate how these topological variations affect three experimentally relevant quantities: the average radius of gyration *R*_g_ of a single bottlebrush polymer within an assembly, the radial distribution function (RDF) between EO and water beads, and the interfacial area *A* covered by the assembled cluster. Please note that the inspection of the equilibrated configurations confirms that, in all systems, the bottlebrush polymers form a single contiguous aggregate at the interface. These structural analyses are well-known to correlate with interfacial activity and packing efficiency of macromolecular surfactants.^64–67^

### Effects of PEG/PS Block Length on Conformation and Interfacial Assembly

Block length is a central topological parameter governing how amphiphilic bottlebrush polymers distribute their hydrophilic and hydrophobic segments at an interface. To isolate this effect, we construct four architectures with increasing block length: the alternating sequence [*E*_4_ *S*_5_]_10_ (block length 1), the short-block topology {[*E*_4_]_2_[*S*_5_]_2_}_5_ (block length 2), the intermediate-block topology {[*E*_4_]_5_[*S*_5_]_5_}_2_ (block length 5), and the diblock architecture [*E*_4_]_10_[*S*_5_]_10_ (block length 10). Figure 2 provides a visual summary of how PEG and PS segments are arranged along the backbone in each topology. These sequence-patterning differences underlie the distinct conformational responses and packing morphologies described below.

Figure 3(a) shows that the radius of gyration, *R*_g_ varies with block length in a nonmonotonic manner, while *R*_g_ decreases from approximately 20 Å for the alternating [*E*_4_ *S*_5_]_10_ to 18.5 Å for the diblock [*E*_4_]_10_[*S*_5_]_10_, the intermediate-block topology {[*E*_4_]_5_[*S*_5_]_5_}_2_ exhibits the lowest *R*_g_. The origin of this behavior is analyzed in detail through the decomposition into tangential and normal components (see Figure 4). Figure 3(b) shows the conformation of the alternating and diblock. The alternating [*E*_4_ *S*_5_]_10_ polymer spreads laterally along the interface, whereas the diblock architecture adopts an L-shaped configuration: the PS-rich block bends upward into the air phase while the PEG-rich block remains hydrated at the interface. The bending of hydrophilic groups toward the interface has also been experimentally reported in phospholipid molecules.^68^

**Figure 3:**
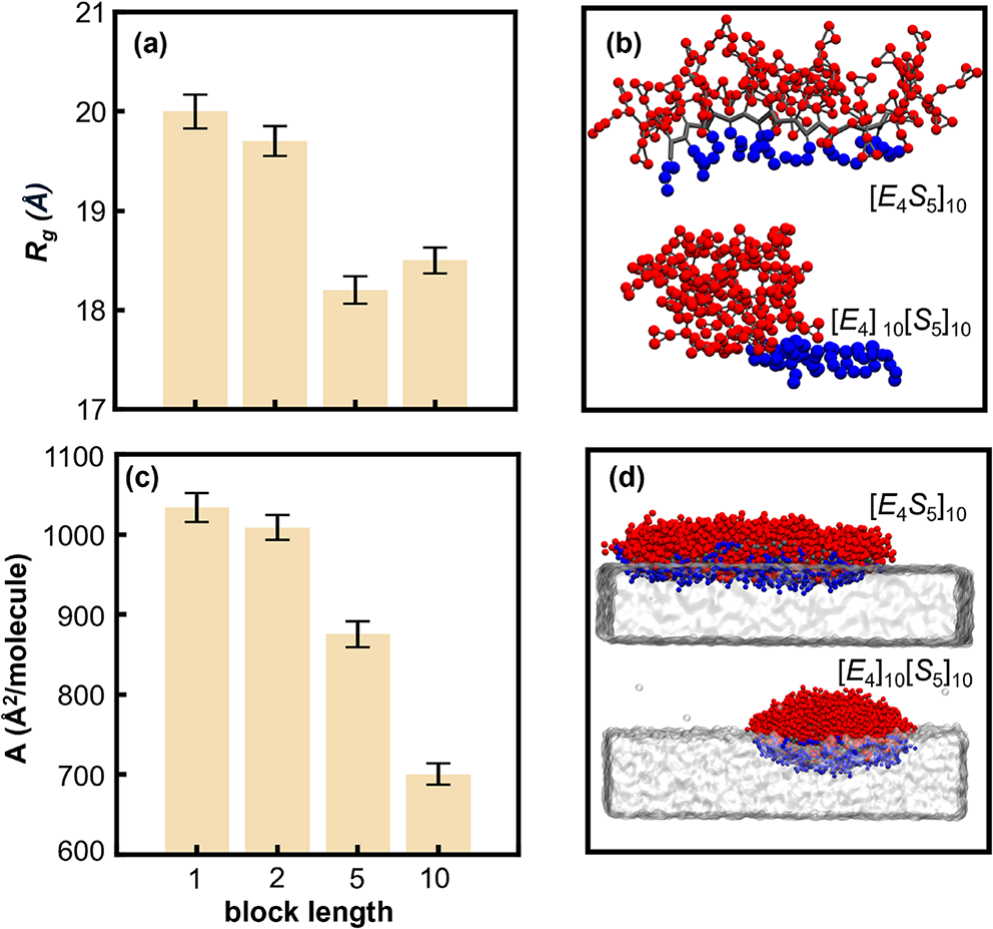
Effect of block length on bottlebrush polymer conformation and interfacial assembly. (a) Radius of gyration *R*_*g*_ for block lengths 1, 2, 5, and 10. (b) Single-chain conformations for the alternating [*E*_4_ *S*_5_]_10_ and diblock [*E*_4_]_10_[*S*_5_]_10_ architectures. (c) Interfacial area per molecule, *A*, averaged over the 20 bottlebrush PEG–PS copolymers with four block-lengths in the assembly. (d) Side-view snapshots of assemblies formed by alternating [*E*_4_ *S*_5_]_10_ and diblock [*E*_4_]_10_[*S*_5_]_10_.

**Figure 4:**
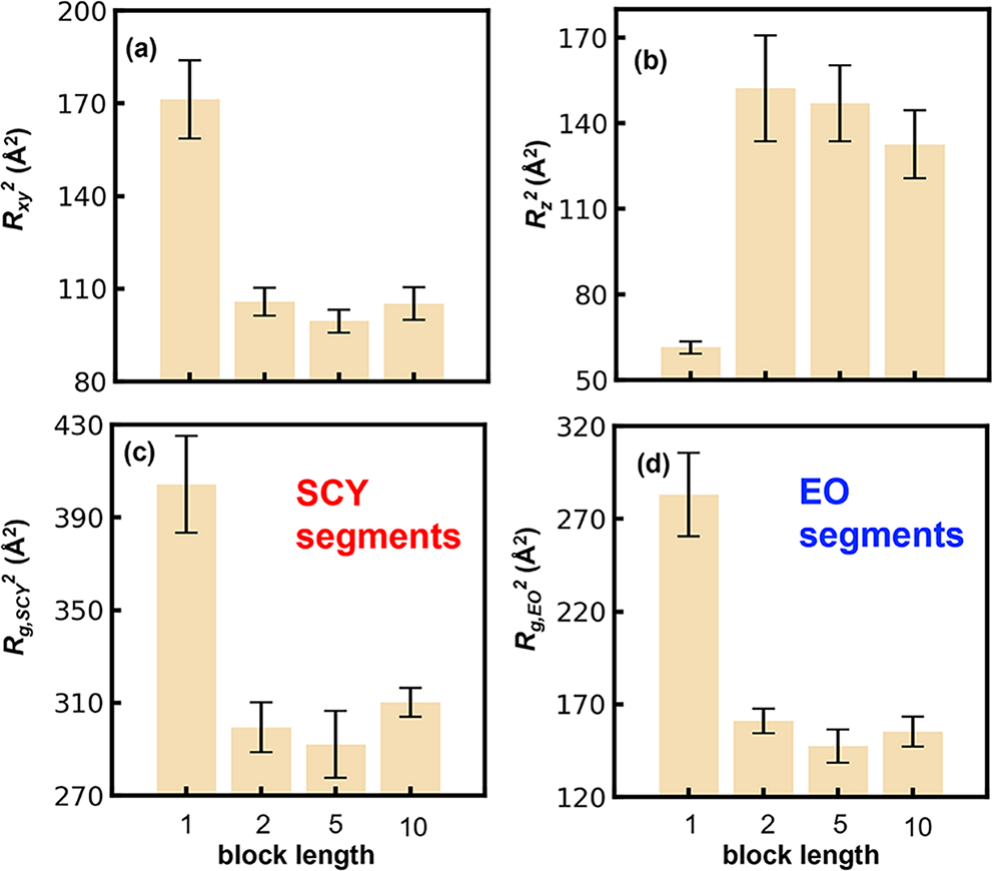
Decomposition of the ⟨*R*_*g*_^2^⟩ as a function of block length. (a) Tangential component ⟨*R*_*xy*_^2^, (b) normal component ⟨*R*_*z*_^2^, (c) contribution from hydrophobic PS (SCY) segments, and (d) contribution from hydrophilic PEG (EO) segments. Error bars represent standard deviations over the sampled configurations.

Besides the chain conformation, the collective assemblies are compared in Figure 3(c,d). The interfacial area per molecule *A* (Å^2^ per molecule) decreases systematically with block length, indicating a transition from extended disk-like aggregates to compact spherical clusters. Side-view snapshots confirm that alternating architectures form broad, low-density assemblies, whereas diblock polymers pack into compact, quasi-spherical aggregates with the PS block oriented toward the air. The emergence of compact diblock assemblies with reduced interfacial covered surface area, therefore, represents a topology-specific mechanism for tuning interfacial packing density and assembly geometry. Our coarse-grained molecular dynamics simulations reveal similar results to a previous study of linear copolymers with diblock and alternating sequences,^69^ where alternating architectures exhibit significantly larger chain dimensions *R*_g_ than diblock copolymers at a low surface concentration. The bent L-shape structures of diblock bottlebrush copolymers and the alignment of alternating along the interface were also observed in linear diblock and alternating copolymers.^69^

To further characterize the anisotropic conformations of bottlebrush polymers at the interface, we decomposed the ⟨*R*_g_^2^⟩ as

1 $$\left\langle R_{g}^{2}\right\rangle =\left\langle R_{g,x}^{2}\right\rangle +\left\langle R_{g,y}^{2}\right\rangle +\left\langle R_{g,z}^{2}\right\rangle$$

and defined the tangential component as

2 $$\left\langle R_{\mathrm{xy}}^{2}\right\rangle =\frac{1}{2}\left(\left\langle R_{g,x}^{2}\right\rangle +\left\langle R_{g,y}^{2}\right\rangle \right)$$

with normal component being ⟨*R*_*z*_^2^⟩ = ⟨*R*_g,*z*_^2^⟩.

As shown in Figure 4, the tangential component ⟨*R*_*xy*_^2^⟩ decreases significantly with increasing block length, from approximately 170 Å^2^ for the alternating architecture (block length 1) to about 100 Å^2^ for block lengths 2–5, followed by a slight increase for the diblock case (block length 10). Figure 4(b) shows the variation in the normal component, ⟨*R*_*z*_^2^⟩. From the figure, it is seen that the alternating polymer (block length 1) has the normal component of 55 Å^2^. However, for a block length of 2, we see a sharp increase in ⟨*R*_*z*_^2^⟩ to 150 Å^2^. It then decreases gradually to 130 Å^2^ with an increase in the block length up to 10. These results indicate that the overall reduction in *R*_g_ with increasing block length is primarily driven by the suppression of lateral spreading along the interface rather than changes in extension perpendicular to it. Furthermore, comparing these two figures shows that it is the tangential component of *R*_g_ which makes the slight increment for the polymer of block length 10, compared to its value of block length 5 (see Figure 3(a)).

To further resolve the structural origin of this behavior, we computed the segment-wise contributions to ⟨*R*_g_^2^⟩ from hydrophobic PS (SCY) and hydrophilic PEG (EO) side chains. As shown in Figure 4(c,d), SCY segments exhibit a decrease in ⟨*R*_g_^2^⟩, from approximately 400 Å^2^ at block length 1 to ∼300 Å^2^ at intermediate block lengths, followed by a slight increase for the diblock case. Similarly, EO segments show a strong reduction, from ∼300 Å^2^ to ∼150 Å^2^, with relatively weak dependence beyond block length 2. These results indicate that the reduction in overall chain size is governed by the relative compaction of both hydrophobic and hydrophilic segments, providing a quantitative explanation for the transition from extended alternating structures to compact diblock assemblies observed in Figure 3.

### Microscopic Origin of Assembly Structures

Figure 3 reflects that the block length governs the conformation and assembled structures of amphiphilic copolymers at the interface. To elucidate the microscopic origin, we take the two extreme cases, the alternating [*E*_4_ *S*_5_]_10_ and diblock [*E*_4_]_10_[*S*_5_]_10_ copolymers, to discuss the effects of the block length. We analyzed the orientational distribution of side chains relative to the interfacial normal, as shown in Figure 5. The angle θ was defined between a side-chain vector and the *z*-axis (normal to the interface), where the side-chain vector was constructed from the backbone reference bead to the center of mass (COM) of the corresponding side chain. This definition provides a direct measure of whether side chains preferentially extend normal to the interface or lie parallel to it, thereby probing how side-chain orientation couples to backbone bending and overall chain compactness.

**Figure 5:**
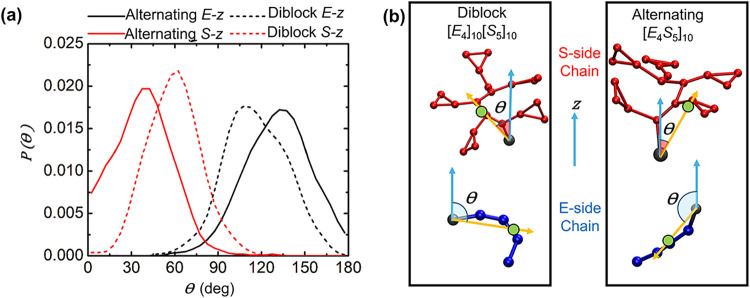
(a) The variation of angles between PEG­(E)/PS­(S) side-chains and normal to the interface (*z*-axis) is plotted for diblock (dashed lines) and alternating assembled system (solid lines). (b) Representative snapshots of the angles defined for PEG/PS side chains with respect to the *z*-axis. The green circle with black borderline shows the actual center-of-mass (COM) of each side chain, the yellow line denotes the vector to the COM with respect to its backbone bead, and the cyan arrow denotes the *z*-direction. All the angle distributions are computed over the last 100 ns of the simulation trajectory. The vector for the side chain is computed with respect to the corresponding backbone beads attached to the side chains and the COM of the side chain.

Figure 5(a) shows the probability distributions of the angle θ between the side-chain vector and the surface normal (*z*-axis) for both hydrophobic (S) and hydrophilic (E) side chains in the diblock architecture. The S side chains exhibit a peak centered around ∼60°, whereas the E side chains peak near ∼115°. Please see the representative snapshot in Figure 5(b). The relatively modest angular separation of ∼50° between these peaks indicates that, although the two types of side chains preferentially occupy different orientational regimes, their distributions still partially overlap. This overlap reflects a cooperative orientational organization in which hydrophobic and hydrophilic side chains adopt complementary, but not strongly opposing, orientations with respect to the interface normal.

In contrast, the alternating architecture displays a markedly different behavior, as shown in Figure 5(a). Here, the S side chains peak at much smaller angles around ∼30°, while the E side chains peak near ∼135°, resulting in an angular separation of nearly 105° (see Figure 5(b) for a representative snapshot). This large separation indicates strong opposite orientations imposed repeatedly along the backbone due to the alternating sequence. As a consequence, hydrophobic and hydrophilic side chains act as distributed and competing orientational constraints, which suppress backbone bending and promote lateral extension along the interface.

The broader orientational separation and reduced overlap between E and S side chains in the alternating architecture lead to enhanced lateral spreading of the alternating bottlebrush copolymer, resulting in a larger radius of gyration and a more loosely packed structure in the assembly. In contrast, the greater overlap and more cooperative orientational organization in the diblock architecture facilitate backbone bending and compact conformations, thereby yielding a lower *R*_g_ and higher packing. These results demonstrate that the sequencing of PEG/PS side chains determines the side-chain and backbone orientations with respect to the interface, thus impacting their chain conformation and aggregated morphologies at the interface.

We also computed the spatial organization of hydrophilic and hydrophobic segments across the interface by obtaining the number-density distributions of EO (PEG) and SCY (in PS) beads along the *z*-direction (see Figure S4 in the SI). For the alternating architecture, the EO and SCY peaks are relatively well-defined and remain localized near their respective preferred regions along the interface normal. In contrast, the diblock architecture exhibits broader distributions with reduced peak separation, with PEG beads extending further into the aqueous phase. This behavior is consistent with both the orientation analysis and the variation in the components of the radius of gyration (Figure 4(a,b)), where the alternating architecture exhibits stronger lateral extension, while the diblock system shows greater elongation along the *z*-direction, leading to a more diffuse density profile.

### Insights into Experimental Results

We next compare our simulation results with recent experimental measurements of interfacial packing and tension for PS–PEG diblock and alternating bottlebrush polymers at the water–air interface reported by Oluwole et al.^29^ In these experiments, surface pressure–area isotherms were obtained by compressing polymer monolayers at the interface, allowing identification of the trough area per molecule at which the system transitions from a gaseous to a liquid-expanded interfacial phase. The authors found that the diblock architecture undergoes this transition at a smaller trough area, approximately 250 nm^2^/molecule, whereas the alternating architecture requires a significantly larger area of about 420 nm^2^/molecule. A larger area at the onset of surface pressure increase indicates that individual polymer molecules occupy a greater interfacial area, consistent with a more extended molecular conformation. This experimental trend closely mirrors our simulation results, which show that alternating architectures possess a larger radius of gyration and occupy a larger interfacial area than diblock polymers (Figure 3). In contrast, the smaller trough area observed experimentally for the diblock polymer reflects more compact conformations and denser interfacial packing, in agreement with the reduced *R*_g_, smaller assembly area, and enhanced orientational ordering identified in our simulations. This is further supported by the orientation distributions obtained from simulations, where the angular separation between hydrophobic and hydrophilic side chains is ∼50° in the diblock system compared to ∼105° in the alternating system, indicating stronger orientational competition and lateral extension in the latter. Thus, the higher packing density is consistent with the lower water/toluene interfacial tensions observed experimentally for diblock polymers. These observations demonstrate a consistent picture in which sequence architecture controls both molecular-scale conformation and macroscopic interfacial packing behavior, and highlight that our simulations provide the molecular-level origin of experimentally observed interfacial transitions that cannot be directly obtained from surface pressure measurements alone.

### Hydration and Local Water Structure as a Function of Block Length

Water molecules at the interface are reorganized in the presence of surfactants.^68,70–72^ To determine how these water molecules are structured around the hydrophilic PEG side chains and how the block length alters the local water structures, we computed the RDF, *g*_EO–W_(*r*) between EO beads and water beads for the four architectures. Physically, *g*_EO–W_(*r*) describes the probability of finding water beads at a distance *r* from EO beads relative to a uniform bulk distribution, and thus characterizes the local hydration structure around the PEG segments. As shown in Figure 6, all topologies display a dominant hydration peak at *r* ≈ 5 Å, corresponding to the first solvation shell around the EO groups. The peak positions remain unchanged, and the peak intensities differ only slightly among the alternating [*E*_4_ *S*_5_]_10_, short-block {[*E*_4_]_2_[*S*_5_]_2_}_5_, intermediate-block {[*E*_4_]_5_[*S*_5_]_5_}_2_, and diblock [*E*_4_]_10_[*S*_5_]_10_ topologies. The inset highlights the small variations in peak height: longer blocks exhibit slightly stronger hydration, while the alternating architecture shows a marginally reduced peak. These differences, however, are minimal compared to the substantial changes in *R*_*g*_ and interfacial packing caused by block-length variation. The results indicate that block length primarily governs the global organization and interfacial occupied area of the bottlebrush polymers, while the local hydration structure around EO segments remains effectively invariant. This invariance reflects the fact that EO beads maintain direct access to the water phase for all topologies, regardless of the degree of intrachain segregation. Thus, block-length–driven morphological transitions arise from changes in intramolecular segregation and backbone conformation, not from changes in EO hydration or solvation-shell structure.

**Figure 6:**
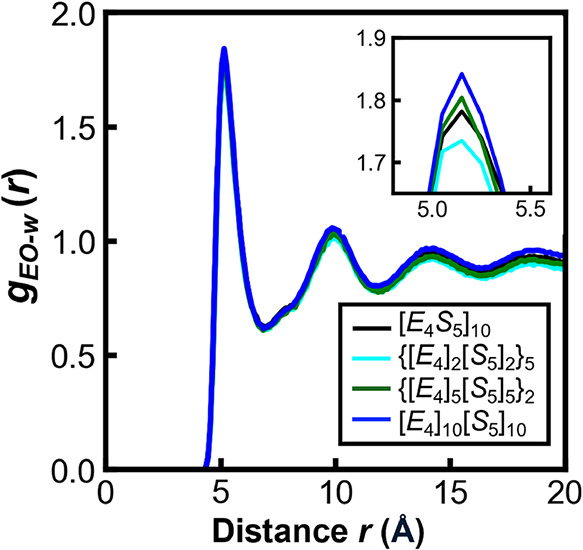
Radial distribution functions between EO beads and water beads for the four block-length architectures. All curves exhibit a first hydration peak near 5 Å. The inset shows a magnified view of the peak region.

### Effects of PEG/PS Side-Chain Dispersity and Grafting Density

Dispersity and grafting density of polymeric side chains widely exist in the synthesized bottlebrush polymers, which play a role in tuning their properties.^73,74^ We next examine the influence of PEG side-chain dispersity *Đ* and grafting density *f*, keeping the total number of EO beads fixed at 40 for all architectures. The structural differences among these topologies are summarized schematically in Figure S1 of the SI, which illustrates how increasing Đ broadens the distribution of PEG side-chain lengths and how decreasing *f* reduces the number of grafted chains per backbone bead.

Figure 7(a,c) shows the effects of PEG side chains, while Figure 7(b,d) focuses on PS side chains. In Figure 7(a), increasing *Đ* of PEG from 1.06 to 1.53 produces only a small change in the *R*_g_, increasing from 18.7 Å to 19.4 Å. This suggests the dispersity does not produce a significant conformational transition in the studied range. Figure 7(c) shows a similarly weak dependence on dispersity in the interfacial area *A*, from 691 to 678 Å^2^/molecule, across the range of *Đ*. These slight variations indicate that ensembles of polymers with different PEG side-chain lengths pack with nearly the same conformation as the bottlebrush copolymers with a uniform side-chain length.

**Figure 7:**
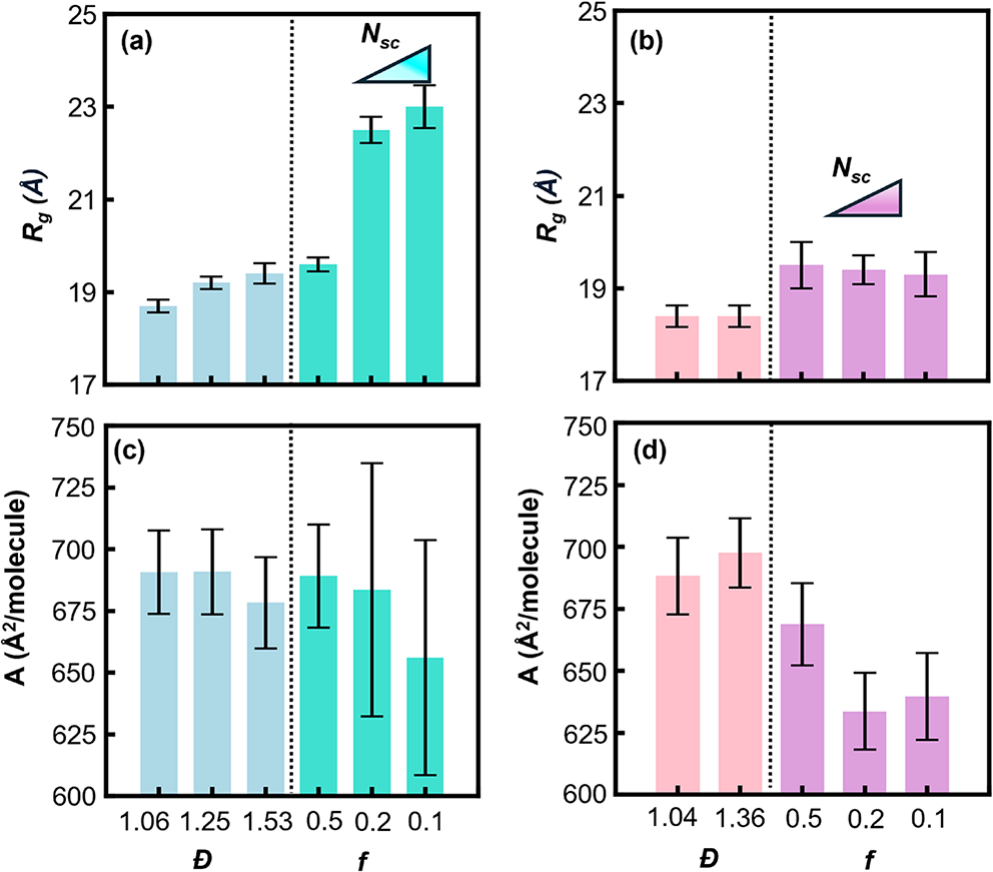
Effect of side-chain dispersity *Đ* and grafting density *f* on polymer conformation and assembled structure is shown here. (a, b) show the variation of the average *R*_g_ for a single polymer chain conformation in the assembly by varying the *Đ*/*f* of PEG and PS, respectively. Corresponding interfacial area per molecule, *A* for PEG and PS is shown in (c, d). In each figure, values to the left of the vertical dashed line correspond to increasing dispersity *Đ*, while values to the right correspond to decreasing grafting density *f*. Note that the triangles in inset (a, b) show that the average side-chain length, *N*_sc_, increases as the grafting density decreases.

In contrast, changing the grafting density *f* produces a much stronger effect. When *f* is reduced from 0.5 to 0.1, *R*_g_ increases drastically from 19.6 to 23 Å (see Figure 7(a)), and the interfacial area per molecule, *A* decreases from 689 to 656 Å^2^/molecule, as shown in Figure 7(c). It is noted that the side-chain length of PEG increases as the *f* decreases. Thus, the increment in *R*_g_ also results from the long PEG side chains. The reduction in the covered area *A* indicates that sparsely grafted long side chains assemble into more compact clusters at the interface despite their larger individual coil sizes. The increased variability in *A* at low *f* reflects the sensitivity of sparsely grafted architectures to the precise spatial arrangement of side chains along the backbone, which allows a broader range of packing configurations. Overall, PEG dispersity introduces only minor changes in polymer conformation and interfacial packing, whereas grafting density, along with the side-chain length, strongly affects both single-chain geometry and collective assembly.

Now, we examine whether variations in PS side-chain dispersity and grafting density exhibit effects similar to those observed for PEG or not. To visualize the variations introduced by PS topology, Figure S2 of SI summarizes the structural differences arising from changes in PS side-chain dispersity *Đ* and grafting density *f*. Figure 7(b) shows that changes in Đ from 1.04 to 1.36 produce similar variations in the radius of gyration *R*_g_, at 18.4 Å. This is similar to the weak dependence observed for PEG dispersity. Figure 7(d) shows that the interfacial area *A* increases slightly from 688 to 698 Å^2^/molecule, indicating less dependence on PS dispersity. This indicates that the dispersity of PS does not strongly affect the conformation and packing of bottlebrush copolymers when the total hydrophobic content is constant.

By varying the PS grafting density, we observed that though the change in *R*_g_ is nominal, the change in area is significant (see Figure 7(d)). Reducing *f* from 0.5 to 0.2 leads to a substantial decrease in *A*, from 669 to 634 Å^2^/molecule reflecting tighter packing of assemblies with fewer hydrophobic side chains. A further reduction to *f* = 0.1 produces only a slight additional change in *A* to 640 Å^2^/molecule.

### Hydration and Packing under PEG Dispersity and Grafting Variations

To determine how PEG topology affects local solvation, we computed radial distribution functions *g*_EO–W_(*r*) between EO beads and water beads for architectures with varying dispersity Đ and grafting density *f*. As shown in Figure 8, changes in *Đ* from 1.06 to 1.53 produce only minor variations in the EO–water RDF, and the height and position of the first hydration peak remain nearly unchanged. This indicates that dispersity, when the total number of EO units is fixed, does not significantly alter the accessibility of EO beads to water or the structure of their local solvation shell. In contrast, reducing the grafting density has a clear effect on hydration. When *f* decreases from 0.5 to 0.1, the first hydration peak increases from about 2.0 to 2.5. Lower grafting density increases the spacing between side chains and reduces steric crowding around the backbone, which exposes a larger fraction of EO beads to the water phase. This is consistent with the larger *R*_g_ values observed at low *f*, where the side chains of PEG adopt more extended conformations that increase the EO–water contact area.

**Figure 8:**
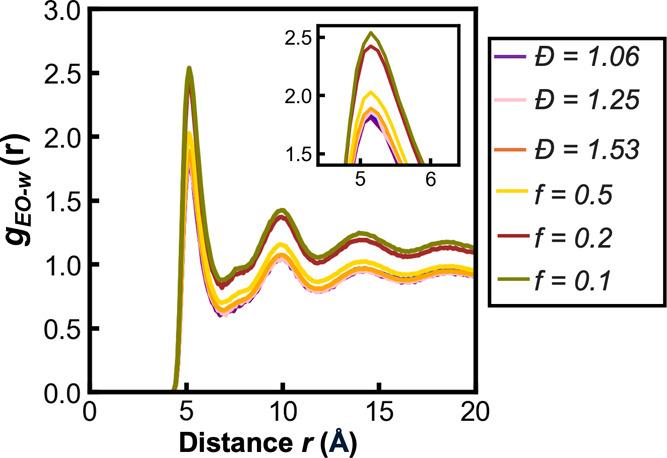
Radial distribution functions *g*_EO–W_(*r*) between EO and water beads for architectures with varying PEG side-chain dispersity *Đ* and grafting density *f*. Dispersity has little effect on EO hydration, while decreasing *f* increases the height of the peak of the first hydration shell.

### Effect of Longer Backbone Length and Higher Surface Concentration on Assembly Structures

To examine the effect of backbone length on the structural properties of the assembly, we performed simulations with *N*_bb_ = 40 for both alternating ([*E*_4_ *S*_5_]_20_) and diblock ([*E*_4_]_20_[*S*_5_]_20_) architectures at the water–air interface. We find that chain expansion occurs in both the alternating and diblock polymer systems; however, the effect is significantly stronger for the diblock system. The overall ⟨*R*_g_^2^⟩ (in Figure S5 and Table S1) increases by ∼53% for the alternating system and by ∼73% for the diblock system, with the latter showing a pronounced enhancement in the normal component (⟨*R*_*z*_^2^⟩, ∼98% increase), indicating stronger elongation along the *z*-direction, whereas the alternating system exhibits comparatively larger lateral expansion (⟨*R*_*xy*_^2^⟩, ∼63% increase). Consistently, the PEG contribution to ⟨*R*_g_^2^⟩ increases from ∼283 to ∼481 Å^2^ (∼70% increase) for the alternating system, compared to a larger increase from ∼155 to ∼436 Å^2^ (∼181% increase) for the diblock system. A complete summary of ⟨*R*_g_^2^⟩ for both 20mer and 40mer systems, along with their decomposition components, is provided in Table S1 of the SI. To further characterize the conformational organization, we analyzed the angular distributions of PEG (E) and PS (S) side chains with respect to the interface normal. For the alternating architecture (see Figure S6a in the SI), the peak positions shift slightly upon increasing backbone length, while maintaining a similar angular separation of ∼100° as that of 20mer. The diblock system also exhibits nearly unchanged peak positions, preserving a smaller separation of ∼50°. However, the angular distributions and peak intensity become strictly sharper and increased, respectively for the diblock architecture. These indicate that increasing backbone length promotes a slightly more laterally extended and flexible conformation in the alternating system, whereas the diblock system adopts a more compact, bent L-shaped configuration.

To investigate how increased interfacial crowding influences molecular packing and orientational organization, we performed additional simulations at higher surface concentration. To do this, the equilibrated aggregates obtained at a 400 × 400 Å^2^ interface were transferred to a reduced interface area of 250 × 250 Å^2^, while maintaining the same box length along the *z*-axis. This reduction in available lateral area imposes a higher degree of interfacial crowding, forcing the assemblies to reorganize under constrained conditions. As shown in Figure S7­(a), both alternating and diblock architectures respond to this increased confinement by expanding laterally (∼18–20%), as reflected in the increase in aggregate area per molecule. The corresponding orientational distributions of PEG (E) and PS (S) side chains are presented in Figure S7­(b). For the alternating system, the peaks remain centered around ∼65° (S) and ∼110° (E), indicating that the strong opposing orientation of hydrophobic and hydrophilic segments is maintained even under increased crowding. In the diblock system, the characteristic separation between S and E segments (with peaks near ∼40° and ∼140°) is similarly conserved, with only minor broadening of the distributions. Overall, these results demonstrate that increasing the surface concentration from the 400 × 400 Å^2^ to the 250 × 250 Å^2^ interface does not lead to a qualitative reorganization of the assemblies. Instead, the systems accommodate the higher packing constraint primarily through lateral expansion, while preserving the intrinsic, architecture-dependent orientational ordering of the side chains.

## Conclusion

In this work, we carried out a systematic coarse-grained molecular dynamics investigation to resolve how molecular topology governs the interfacial assembly of amphiphilic bottlebrush polymers at a water/air interface. By independently varying block length, side-chain dispersity, and grafting density while holding the overall chemical composition fixed, we quantified their effects on single-chain conformation, side-chain orientation, hydration, and collective packing. Among these parameters, block length exerts the strongest control over assembly. Increasing block length leads to an overall reduction in the radius of gyration, a decrease in interfacial area per molecule, and a transition from extended, surface-parallel conformations to compact bent configurations. In the extreme diblock limit, the bottlebrush adopts a distinct L-shaped configuration, with hydrophobic PS side chains preferentially aligned along the surface normal toward the air phase and hydrophilic PEG side chains remaining laterally oriented and hydrated at the interface. This architecture-dependent conformational asymmetry leads to dense packing and reduced interfacial surface area.

Side-chain dispersity plays a comparatively minor role in the studied range. When the total number of EO or PS units is fixed, increasing dispersity produces only small changes in chain dimensions and interfacial area, along with increasing the EO-water coordination. In contrast, grafting density along with side-chain lengths strongly modulates both single-chain geometry and collective assembly. Reducing PEG grafting density increases backbone flexibility, and expands single-chain dimensions, yet assemblies become slightly more compact. For PS, lowering grafting density primarily affects hydrophobic packing at the interface, leading to tighter assemblies with relatively low configurational variability. These results demonstrate that grafting density and block length act as independent but complementary controls, while dispersity alone is insufficient to drive qualitative changes in interfacial morphology under the conditions studied. Additionally, extending the backbone length reveals that PEG segments exhibit increased spatial extension, indicating enhanced segmental stretching. Furthermore, increasing surface concentration by 2.5 times does not induce a significant change in the side-chain orientation, but it enhances the lateral expansion of both alternating and diblock copolymers.

The present study focuses on 16 manually designed architectures. An important direction for future work is to apply machine learning to explore a large design space of bottlebrush copolymers to establish the relationship between architectures and assembly. This will extend the current understanding and provide more general guidelines for designing bottlebrush copolymers. Moreover, extending the backbone length and exploring a broader range of surface concentrations would provide a more complete understanding of how molecular architecture and crowding collectively influence the structural properties and orientational organization in bottlebrush assemblies.

## Supplementary Material



## Notes

Materials contain Figures S1 and S2: showing the bottlebrush polymer architectures that have been used in our work to investigate the role of side-chain dispersity and grafting density of PEG and PS polymer, respectively; It also includes Figure S3, which describe the method used to determine the interfacial area *A* occupied by the polymer assemblies; furthermore, Figure S4 present the number-density distributions of PEG and PS components along the interface normal; Figures S5 and S6 along with Table S1 provide additional analysis of the effect of increased backbone length; Figure S7 illustrate the effect of increased surface concentration (PDF)
