# Supplementary material for: Topological Effects of Bottlebrush Copolymer on Their Assembly at the Water/Air Interface
Source: Langmuir. 2026 May 28;42(22):15466–77. doi: 10.1021/acs.langmuir.6c00890 (PMC13262039; doi:10.1021/acs.langmuir.6c00890)
Supplement: Supplementary file 1 [file la6c00890_si_001.pdf]

# Supporting Information: Topological effects of bottlebrush copolymer on their assembly at the water/air interface

Shubhadeep Nag, Nazrul Islam, Titilayo Deborah Oluwole, Jimmy Lawrence, and Yaxin An*

*Department of Chemical Engineering, Louisiana State University, Baton Rouge, LA, 70803*

E-mail: yxan@lsu.edu

Phone: +1 225-578-0721

Number of pages: 10

Number of figures: 7

Number of tables: 1

**Table of Contents**

| Section | Pages |
|---|---|
| Side-chain dispersity and grafting density on bottlebrush architectures (Figures S1, S2) | S2-S3 |
| Analysis (Figure S3) | S3-S4 |
| Number-density distribution along the interface normal (Figure S4) | S4-S7 |
| Assembly structure of increased backbone length (Figures S5, S6; Table S1) | S7-S8 |
| Effect of increased surface concentration on side-chain orientation (Figure S7) | S9-S10 |

# Side-Chain Dispersity and Grafting Density on Bottlebrush Architectures

Figures S1 and S2 illustrate the model bottlebrush polymer architectures used to investigate the role of side-chain dispersity and grafting density. Figure S1 presents schematic representations of PEG side-chain architectures, where increasing dispersity broadens the distribution of side-chain lengths while preserving the underlying backbone structure, and decreasing grafting density reduces the number of grafted PEG chains, increasing the spacing between grafting sites.

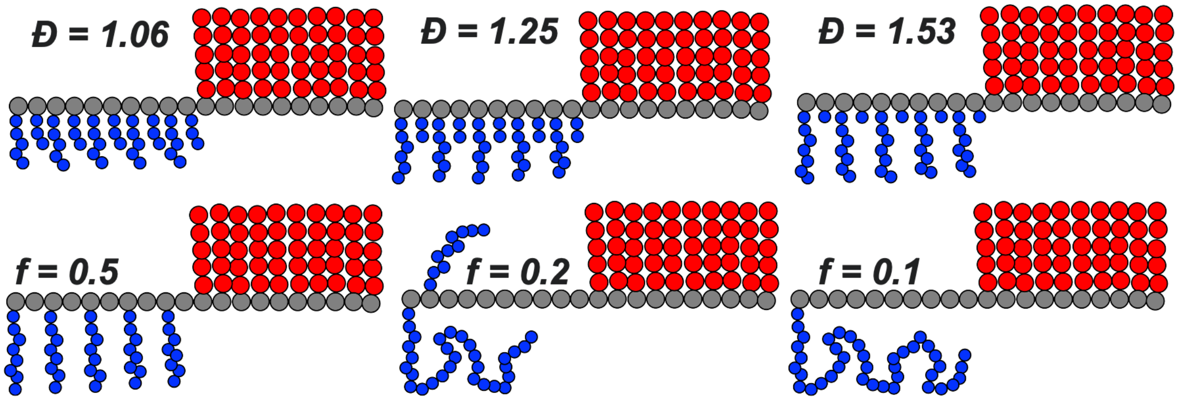


Figure S1: Schematic representation of PEG side-chain architectures. Top row: increasing dispersity, $Đ = 1.06$, 1.25, 1.53 broadens the range of PEG side-chain lengths. Bottom row: decreasing grafting density, $f = 0.5, 0.2, 0.1$ reduces the number of grafted PEG chains and increases the spacing between grafting points. PEG side chains are shown in blue, PS in red, and backbone beads in gray.

Figure S2 shows representative conformations highlighting analogous variations in PS side-chain dispersity and grafting density, illustrating the resulting changes in side-chain length heterogeneity and chain spacing along the backbone.

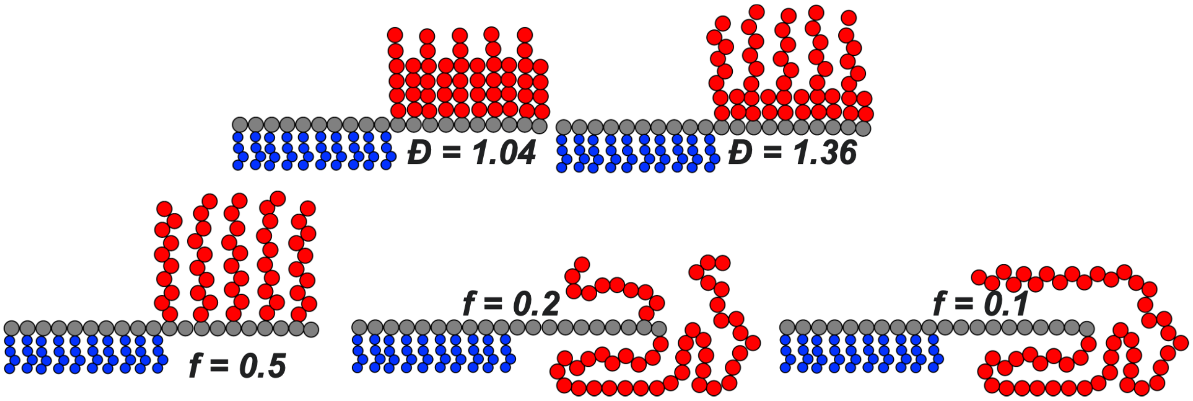


Figure S2: Representative conformations of bottlebrush polymers illustrating the effects of PS side-chain dispersity and grafting density. Top row: increasing dispersity from $Đ$ = 1.04 to 1.36 increases variation in side-chain lengths while maintaining the overall brush morphology. Bottom row: decreasing grafting density from $f = 0.5$ to 0.1 reduces the number of grafted PS chains and yields more flexible, collapsed conformations. Red beads denote PS, blue beads denote PEG, and gray beads represent the backbone.

# Analysis

In this work, the radial distribution function $g_{\mathrm{EO-W}}(r)$ is defined as:

$$g_{\mathrm{EO-W}}(r) = \frac{1}{4\pi r^2 \rho_{\mathrm{W}} N_{\mathrm{EO}}} \left\langle \sum_{i=1}^{N_{\mathrm{EO}}} \sum_{j=1}^{N_{\mathrm{W}}} \delta\left(r - r_{ij}\right) \right\rangle, \tag{S1}$$

where $N_{\mathrm{EO}}$ and $N_{\mathrm{W}}$ are the total number of EO beads and water beads, respectively, $r_{ij}$ is the distance between EO bead $i$ and water bead $j$, and $\rho_{\mathrm{W}}$ is the number density of water beads. The angular brackets denote an ensemble average over configurations. This function represents the probability of finding water beads at a distance $r$ from EO beads relative to an ideal uniform distribution, and thus characterizes the local hydration structure around the PEG segments.

We also computed the radius of gyration $R_g$ of each bottlebrush polymer. It is defined as:

$$R_g = \left( \frac{1}{N} \sum_{i=1}^{N} \left| \mathbf{r}_i - \mathbf{r}_{\mathrm{cm}} \right|^2 \right)^{1/2}, \tag{S2}$$

where $N$ is the total number of beads in the polymer (including backbone and side chains),

$\mathbf{r}_i$ is the position of bead $i$, and $\mathbf{r}_{\mathrm{cm}}$ is the center-of-mass position of the polymer. This definition accounts for both in-plane spreading at the interface and out-of-plane extension of side chains.

To quantify the interfacial area $A$ occupied by the polymer assemblies, all polymer beads were projected onto the $xy$ plane. The projected bead positions were then used to construct the smallest two-dimensional polygon (convex hull) enclosing all points. The interfacial area was defined as the area of this convex hull, representing the maximal lateral area of the polymer assembly at the interface, as illustrated in Fig. S3. Since the bottlebrush polymers form a single contiguous aggregate, the computed area corresponds to the total cluster area. Thus, to enable comparison across different systems employed in our work, we report the interfacial area per molecule, $A$. It is therefore defined as

$$A = \frac{Total\ Cluster\ Area}{Number\ of\ Molecules} \tag{S3}$$

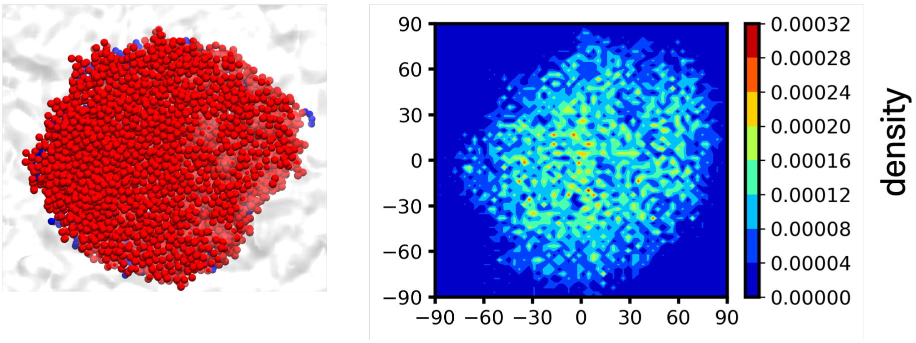


Figure S3: Visualization of the polymer amphiphile assembly (left) and the corresponding two-dimensional surface density map used to quantify the total cluster area coverage of polymer surfactants at the interface (right).

## Number-density distribution along the interface normal

To quantify the spatial distribution of the different components across the water–air interface, we computed the number-density distributions of PEG (E) and PS (S) segments along the interface normal ($z$-direction). For each frame of the trajectory, the centers of mass (COMs)

of the PEG and PS side chains were calculated. The $z$-coordinates of these COMs were then shifted relative to a fixed reference position ($z_0 = -100$ Å) and binned along the $z$-axis using uniform bins of width $\Delta z$. The resulting histograms were accumulated over all frames and averaged over the trajectory. The number-density profile is defined as

$$\rho(z) = \frac{\langle N(z) \rangle}{\Delta z} \tag{S4}$$

where $\langle N(z) \rangle$ is the average number of side-chain COMs (PEG or PS) in a bin centered at position $z$, and $\Delta z$ is the bin width. Thus, $\rho(z)$ represents the average number of PEG or PS segments per unit length along the $z$-direction, providing a one-dimensional number-density profile that captures the relative spatial distribution of hydrophilic and hydrophobic components across the interface.

The choice of a fixed reference shift ($z_0$) provides a consistent coordinate system across all frames and systems. Although the instantaneous position of the water–air interface may fluctuate slightly during the simulation, these fluctuations are small compared to the bin size and the overall extent of the system. Therefore, using a single reference value for all frames does not affect the qualitative features of the density profiles and allows for direct comparison between different architectures.

The density profiles show that, for the alternating architecture, the EO peak is located near $\sim 0 - 2$ Å, while the SCY peak appears at $\sim 15 - 18$ Å, indicating a pronounced spatial separation along the interface normal. The peaks are also relatively sharp, suggesting that both hydrophilic and hydrophobic segments remain confined close to their preferred interfacial positions. In contrast, for the diblock system, the EO peak shifts to $\sim -8$ to $-10$ Å and the SCY peak is located at $\sim$ 12–15 Å, with broader distributions and reduced peak separation, indicating a more diffuse spatial distribution along the $z$-direction.

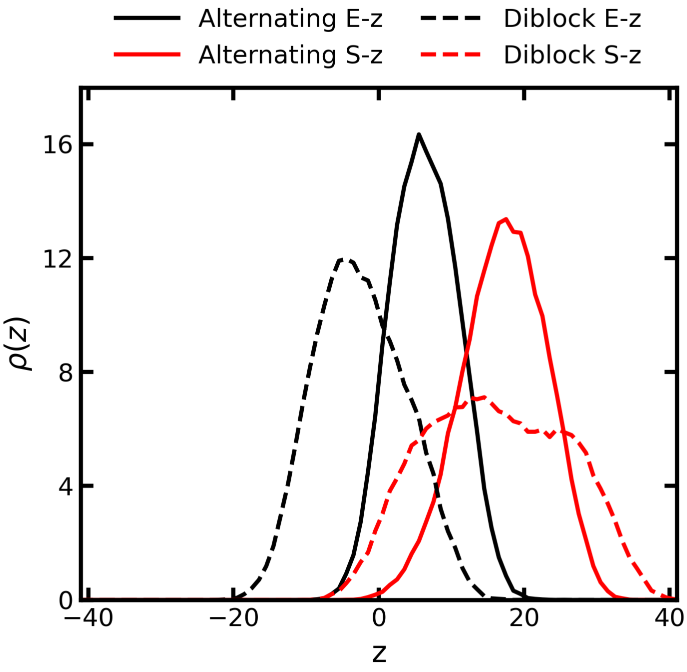


Figure S4: Number-density profiles of PEG (E) and PS (S) beads along the interface normal ($z$-axis) for alternating (solid lines) and diblock (dashed lines) architectures.

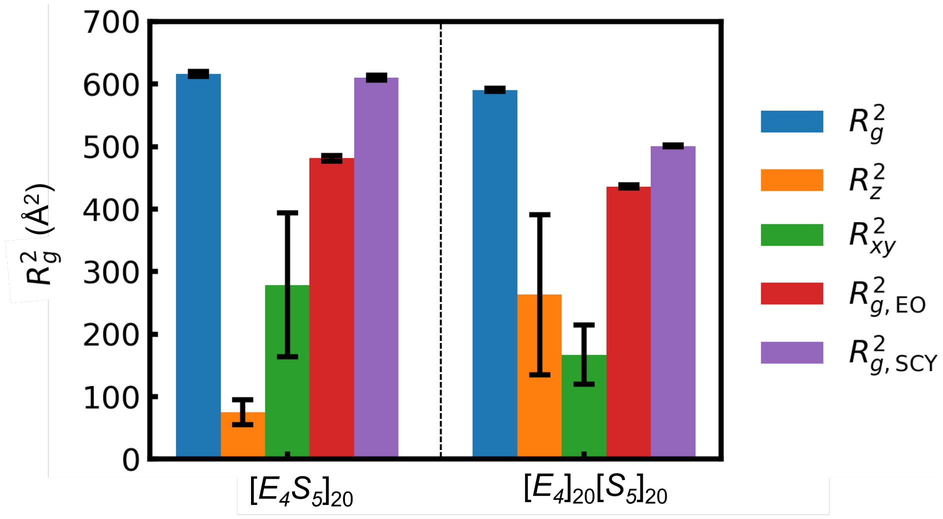


Figure S5: Values of $\langle R_g^2 \rangle$, along with its normal ($\langle R_z^2 \rangle$) and tangential ($\langle R_{xy}^2 \rangle$) components, and segment-wise contributions ($R^2_{g,\mathrm{EO}}$ and $R^2_{g,\mathrm{SCY}}$) are shown here for alternating ($[E_4S_5]_{20}$) and diblock ($[E_4]_{20}[S_5]_{20}$) assembly structure. Error bars represent standard deviations over sampled configurations.

Table S1: Comparison of structural properties for 20-mer and 40-mer bottlebrush polymers. Percentage change is reported relative to the 20-mer.

| Alternating, $[E_4S_5]_{20}$ | | | |
|---|---|---|---|
| | 20-mer | 40-mer | Change (%) |
| $R_g^2$ (Å$^2$) | 400.20 | 616.03 | +53.4 |
| $R_z^2$ (Å$^2$) | 61.38 | 74.92 | +22.1 |
| $R_{xy}^2$ (Å$^2$) | 171.22 | 278.62 | +62.7 |
| $R_{g,\mathrm{EO}}^2$ (Å$^2$) | 283.17 | 481.32 | +70.0 |
| $R_{g,\mathrm{SCY}}^2$ (Å$^2$) | 404.20 | 610.16 | +51.0 |
| Diblock, $[E_4]_{20}[S_5]_{20}$ | | | |
| | 20-mer | 40-mer | Change (%) |
| $R_g^2$ (Å$^2$) | 341.14 | 590.98 | +73.2 |
| $R_z^2$ (Å$^2$) | 132.61 | 263.13 | +98.4 |
| $R_{xy}^2$ (Å$^2$) | 105.15 | 167.01 | +58.8 |
| $R_{g,\mathrm{EO}}^2$ (Å$^2$) | 155.31 | 436.09 | +180.8 |
| $R_{g,\mathrm{SCY}}^2$ (Å$^2$) | 310.11 | 501.19 | +61.6 |

# Assembly structure of increased backbone length

To examine the effect of increasing backbone length, simulations were performed for both alternating and diblock architectures with $N_{bb} = 40$. The system setup, interaction parameters, and simulation protocol were identical to those used for the $N_{bb} = 20$ systems, ensuring direct comparability. The corresponding conformational changes are quantified through the $\langle R_g^2 \rangle$ and its components, as shown in Fig. S5. For the alternating architecture ($[E_4S_5]_{20}$), the overall radius of gyration $\langle R_g^2 \rangle$ increases by $\sim 53\%$, with a larger contribution from the tangential component ($\langle R_{xy}^2 \rangle$, $\sim 63\%$ increase) compared to the normal component ($\langle R_z^2 \rangle$, $\sim 22\%$ increase), indicating predominantly lateral expansion along the interface. In contrast, the diblock architecture ($[E_4]_{20}[S_5]_{20}$) exhibits a stronger increase in overall size ($\sim 73\%$), accompanied by a pronounced enhancement in the normal component ($\langle R_z^2 \rangle$) ($\sim 98\%$ increase) compared to an increment of $\sim 59\%$ in the tangential component, suggesting significant elongation along the $z$-direction.

Consistently, the segment-wise contributions to $\langle R_g^2 \rangle$ show that the PEG component increases from $\sim 283$ to $\sim 481$ Å$^2$ ($\sim 70\%$ increase) for the alternating system, compared

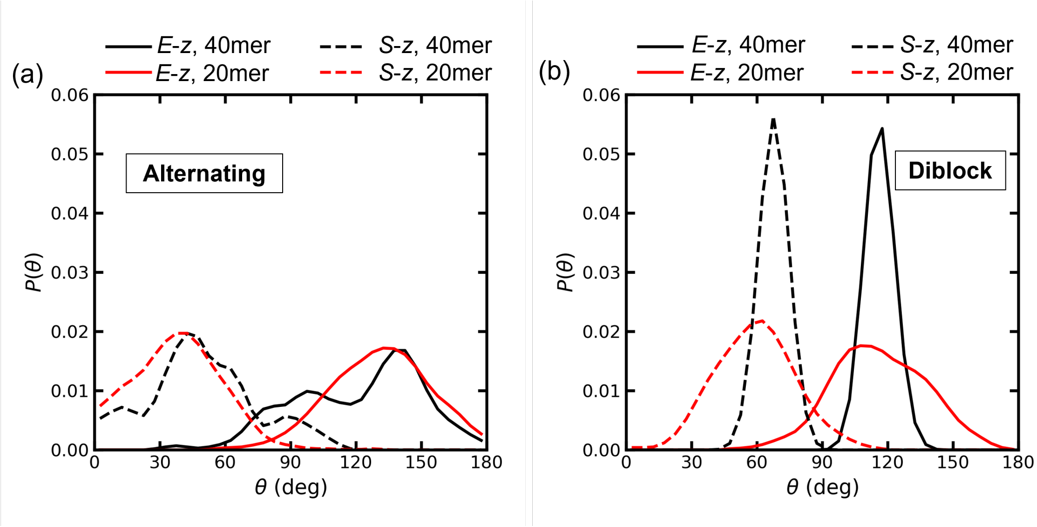


Figure S6: Probability distributions of the angle $\theta$ between PEG (E) and PS (S) side chains and the interface normal (z-axis) for (a) alternating and (b) diblock architectures, comparing backbone lengths of 20-mer (red) and 40-mer (black). Solid lines correspond to E segments and dashed lines correspond to S segments.

to a substantially larger increase from $\sim 155$ to $\sim 436$ Å$^2$ ($\sim 181\%$ increase) for the diblock system. The SCY contribution also increases in both cases, although more moderately ($\sim 51\%$ and $\sim 62\%$ for alternating and diblock, respectively).

To further understand the conformational organization, we analyzed the angular distributions of PEG (E) and PS (S) side chains with respect to the interface normal. For the alternating architecture, the angular distributions for 20-mer and 40-mer exhibit similar peak positions, with S and E peaks located at $\sim 30$–$40°$ and $\sim 135$–$140°$, respectively, and show comparable intensity profiles, indicating minimal change in orientational organization with increasing backbone length. In contrast, for the diblock architecture, the peak positions remain similar between 20-mer and 40-mer (S $\sim 60$–$70°$, E $\sim 115$–$120°$), but the distributions become significantly sharper at 40-mer, as reflected by the increased peak height and reduced width, indicating a more well-defined orientation.

## Effect of Increased Surface Concentration on Side-Chain Orientation

To understand the effect of higher surface concentration, we performed additional simulations by placing equilibrated aggregates obtained at the $400 \times 400$ Å$^2$ interface, i.e. $2.08\times10^{-28}$ mol per Å$^2$ into a smaller $250 \times 250$ Å$^2$ interfacial area, i.e. $5.31\times10^{-28}$ mol per Å$^2$ for both alternating and diblock architectures, while keeping the box length along the $z$-axis constant. Under increased surface crowding, the aggregate area per molecule for the alternating architecture, and for the diblock architecture, increases by 18-20%, indicating lateral expansion of the aggregates (see Figure S7a).

The corresponding orientational distributions of PEG (E) and PS (S) side chains with respect to the interface normal are shown in Fig. S7b along with those obtained for the $400 \times 400$ Å$^2$ system. From the figure, it is evident that for both architectures, the peak positions and their intensities remain largely unchanged compared to those obtained for the $400 \times 400$ Å$^2$ system (see corresponding discussion in Microscopic origin of assembly structures of the main manuscript). These results indicate that, despite the increase in interfacial area, the characteristic orientational separation between hydrophobic and hydrophilic segments remains unchanged.

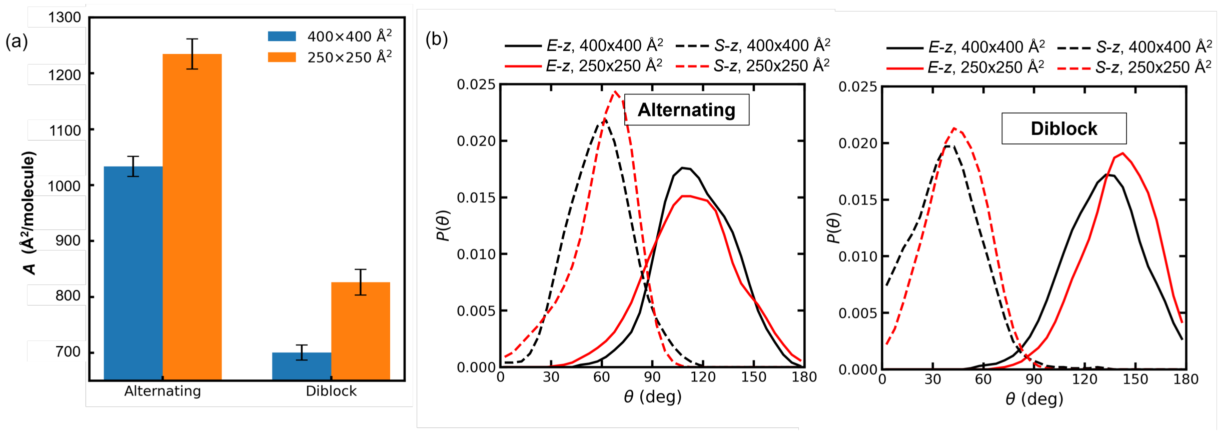


Figure S7: (a) Interfacial area per molecule $A\,(\mathring{A}^2/\text{molecule})$ for alternating and diblock architectures at $400 \times 400$ $\mathring{A}^2$ and $250 \times 250$ $\mathring{A}^2$ interfaces. Error bars represent standard deviations. (b)Probability distributions of the angle $\theta$ between PEG (E) and PS (S) side chains and the interface normal (z-axis) for alternating (left) and diblock (right) architectures at both surface concentrations.
